# Glycome Profiling and Bioprospecting Potential of the Himalayan Buddhist Handmade Paper of Tawang Region of Arunachal Pradesh

**DOI:** 10.3389/fpls.2022.831589

**Published:** 2022-05-23

**Authors:** Muzamil Ahmad Rather, Anutee Dolley, Nabajit Hazarika, Vimha Ritse, Kuladip Sarma, Latonglila Jamir, Siddhartha Shankar Satapathy, Suvendra Kumar Ray, Ramesh Chandra Deka, Ajaya Kumar Biswal, Robin Doley, Manabendra Mandal, Nima D. Namsa

**Affiliations:** ^1^Department of Molecular Biology and Biotechnology, Tezpur University, Napaam, India; ^2^Department of Environmental Biology and Wildlife Sciences, Cotton University, Guwahati, India; ^3^Department of Environmental Science, Nagaland University, Lumami, India; ^4^Department of Zoology, Cotton University, Guwahati, India; ^5^Department of Computer Science and Engineering, Tezpur University, Napaam, India; ^6^Centre for Multi-disciplinary Research, Tezpur University, Napaam, India; ^7^Department of Chemical Sciences, Tezpur University, Napaam, India; ^8^Department of Biochemistry and Molecular Biology and Complex Carbohydrate Research Center, University of Georgia, Athens, GA, United States

**Keywords:** *Daphne papyracea*, handmade paper, Arunachal Pradesh, glycome profiling, species distribution modeling

## Abstract

The paper and pulp industry (PPI) is one of the largest industries that contribute to the growing economy of the world. While wood remains the primary raw material of the PPIs, the demand for paper has also grown alongside the expanding global population, leading to deforestation and ecological imbalance. Wood-based paper production is associated with enormous utilization of water resources and the release of different wastes and untreated sludge that degrades the quality of the environment and makes it unsafe for living creatures. In line with this, the indigenous handmade paper making from the bark of *Daphne papyracea*, Wall. ex G. Don by the Monpa tribe of Arunachal Pradesh, India is considered as a potential alternative to non-wood fiber. This study discusses the species distribution modeling of *D. papyracea*, community-based production of the paper, and glycome profiling of the paper by plant cell wall glycan-directed monoclonal antibodies. The algorithms used for ecological and geographical modeling indicated the maximum predictive distribution of the plant toward the western parts of Arunachal Pradesh. It was also found that the suitable distribution of *D. papyracea* was largely affected by the precipitation and temperature variables. Plant cell walls are primarily made up of cellulose, hemicellulose, lignin, pectin, and glycoproteins. Non-cellulosic cell wall glycans contribute significantly to various physical properties such as density, crystallinity, and tensile strength of plant cell walls. Therefore, a detailed analysis of non-cellulosic cell wall glycan through glycome profiling and glycosyl residue composition analysis is important for the polymeric composition and commercial processing of *D. papyracea* paper. ELISA-based glycome profiling results demonstrated that major classes of cell wall glycans such as xylan, arabinogalactans, and rhamnogalacturonan-I were present on *D. papyracea* paper. The presence of these polymers in the Himalayan Buddhist handmade paper of Arunachal Pradesh is correlated with its high tensile strength. The results of this study imply that non-cellulosic cell wall glycans are required for the production of high-quality paper. To summarize, immediate action is required to strengthen the centuries-old practice of handmade paper, which can be achieved through education, workshops, technical know-how, and effective marketing aid to entrepreneurs.

## Introduction

Worldwide consumption of paper has grown intensely, and papermaking has been showing a continuously increasing trend. The pulp and paper industry is one of the largest industries predominated by East Asian, North American, and Northern European countries and makes a major contribution to the world’s economy as well as livelihoods (Bajpai, 2018). China and India are reported to become the key countries in terms of paper production globally. It has been reported that about 371 and 399 million metric tons of paper were consumed globally in 2009 and 2020, respectively. It is expected that it would reach up to 461 million metric tons by 2030 (Ian Tiseo, 2021). The average consumption of paper per year per individual is recorded as 265, 7, and 40 kg by Americans, Africans, and Asians, respectively. The global average consumption of paper is 55 kg/individual/year while Japan consumes 215 kg, Finland (194 kg), China (65 kg), and India (9 kg) (Bajpai, 2013). On the other hand, wood-based paper production is associated with enormous utilization of wood and water resources, and release of different wastes and untreated sludge, thus degrading the quality of the environment and making it unsafe for living creatures. Different toxic effluents are released during pulping and bleaching of woods, including chlorine compounds, chlorinated organics, furans, and dioxins that are collectively responsible for water, air, and land pollution (Singh and Chandra, 2019). Therefore, paper-producing industries are under constant environmental, economic, and political pressures to minimize the use of chemical processes to decrease the volume and toxicity of industrial wastewaters. So, to minimize the utilization of enormous supply of energy and chemicals, innovative approaches should be focused to achieve ecosystem sustainability during paper production (Bajpai, 2012). In this regard, papermaking from non-wood fibers is considered a potential alternative to meet the limitations and environmental cues associated with wood-based papermaking. Non-wood fibers or alternate fibers are non-woody cellulosic plant materials. These plants are usually annual, and the cellulosic fibers obtained from them are processed and developed into papers. Some of the globally used non-wood papermaking plants include sugar cane bagasse, kenaf, straws, hemp, jute, bamboo, sisal, cotton linters, reeds, and abaca (Abd El-Sayed et al., 2020). Khakifirooz et al. (2012) studied the chemical properties and morphological characteristics of *Hibiscus cannabinus* to validate its utility for papermaking. They demonstrated the presence of holocellulose, alpha-cellulose, and lignin with a percentage of 72.31, 48.2, and 16.27%, respectively, in *H. cannabinus* fibers. Furthermore, fiber length (2,553 μm), width (22.35 μm), width of lumen (5.23 μm), and cell wall thickness (11.90 μm) were also demonstrated, which indicates that it could be used as a potential source of raw material for the papermaking industry.

Handmade papermaking is regarded as an environmentally friendly, energy-efficient, and promising approach. The industry has a high potential for energy conservation, environment protection, and promotion of local entrepreneurship. Traditionally, handmade papers are made from wood-free materials, like silk cotton, banana fibers, cotton, kenaf, mat grass, bagasse, and agro-wastes. It has been reported that handmade papermaking was earlier practiced in China but due to its high potential, it is also being practiced by many European and Asian countries as well (Jain and Gupta, 2021). Handmade papermaking is regarded as the most important environment-friendly approach to paper production as it emphasizes the conservation of natural resources like forests, large trees, etc., along with the significant reduction of detrimental compounds leaking into the environment. Bidin et al. (2015) discussed the suitability of five aquatic plant fibers, *viz*., *Cyperus digitatus*, *Cyperus halpan*, *Cyperus rotundus*, *Scirpus grossus*, and *Typha angustifolia*, for handmade papermaking. The chemical composition and fiber dimensions of each plant were studied. It was demonstrated that long (0.71–0.83 mm), thin plant fibers (9.13–12.11 μm) were observed in all the tested plant fibers with narrow lumen (4.32–7.30 μm) and thin cell wall (2.25–2.83 μm). Furthermore, slenderness ratio, flexibility coefficient, and low Runkel ratio ranged from 73.77 to 89.34, 0.84 ± 0.17, and 52.91 to 58.08, respectively. Therefore, based on physical properties, fiber characteristics, and chemical composition, it was concluded that *S. grossus*, *C. rotundus*, and *T. angustifolia* are suitable candidates for handmade papermaking. Similarly, Mejouyo et al. (2020) discussed the production and characterization of biodegradable handmade paper obtained from *Sida rhombifolia* plant cellulose. The paper was prepared by a traditional method called “Kraft method,” and thereafter, physical characterization was evaluated, *viz*., tensile properties (Young’s modulus, elongation at break, and stress at break), water, moisture absorption rate, etc.

*Daphne papyracea* Wall. ex G. Don is a branched, erect evergreen shrub belonging to the *Thymelaeaceae* family. It is native to Arunachal Pradesh, Assam, China South-Central, China Southeast, Bangladesh, Myanmar, West Himalaya, Nepal, and Pakistan (Moshiashvili et al., 2020). It propagates in cool lime-free well-drained sandy loam, tolerates a dip in temperature up to −10°C, and prefers shady places but grows even in partial sunny places in forested areas (coniferous as well as broad-leaved) at an elevation of 700-3,200 m. It is propagated mostly through seed germination, while its propagation through stem cuttings and root suckers is also reported (Sharma and Devi, 2013). The plant has dull green narrow-lanceolate to oblanceolate leathery leaves, white-greenish flowers that blossom from November to April, gray bark, and orange to deep red fruits (Sovrlic and Manojlovic, 2017). In Arunachal Pradesh, the plant is locally known as “*Shukshing*” and is being used traditionally for making papers by the *Monpa* tribe of Tawang and West Kameng regions of Arunachal Pradesh. The inner fibrous bark of the plant is an important source of handmade paper. The thickness and length of the *D. papyracea* fibers are categorically more than that of the other common handmade papers. The thickness and the length of the rice straw, sugarcane, and bamboo fibers have been reported as 8.8 mm and 1.5 mm, 20 mm and 1.7 mm, and 9.14 mm and 1.09-2.33 mm, respectively, which are significantly lesser than that of *D. papyracea* fibers (6-20 mm and 2-12 mm) (Paul et al., 2006).

The complexity, heterogeneity, and variability of the plant cell wall, which is due to ultra-crosslinking of cellulose microfibers embedded in a macromolecular matrix of glycans, impede the high-throughput analysis of plant cell wall structure and composition. The emergence of glycome profiling, a comprehensive collection of glycan-directed monoclonal antibodies, is an important tool for the rapid and semi-quantitative identification of the major non-cellulosic glycans present in plant cell walls (Ruprecht et al., 2020). The significant variations in the cell types, organs, age, developmental stage, and growth environment depending on both biotic and abiotic stress can also be determined by glycome profiling (Pattathil et al., 2017; Ruprecht et al., 2020). Thus, glycome profiling provides an effective approach to understand plant cell wall composition, structure, and architecture to overcome the resistance of cell walls to biological or chemical catalysts (Pattathil et al., 2015). This work is focused on the habitat suitability or species distribution modeling of *D. papyracea* in Arunachal Pradesh, methods for the production of the paper from the inner bark of the plant, and glycome profiling of the paper by plant cell wall glycan-directed monoclonal antibodies (mAbs).

## Materials and Methods

### Habitat Suitability or Species Distribution Modeling for *Daphne papyracea* in Arunachal Pradesh

#### Study Area

**FIGURE 1:**
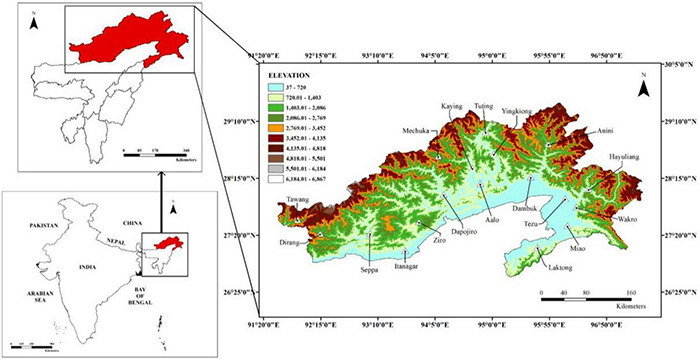
Location map of the state of Arunachal Pradesh depicting the elevation gradient (in meters).

Arunachal Pradesh is situated at 91°54′E to 97°05′E longitudes and 26°50′E-29°55′E latitudes in the northeastern part of India (Figure 1). It is bordered by Tibet to the North, Myanmar to the East, Bhutan to the West, and Assam to the South. It has an annual rainfall ranging from 2,000 to 5,000 mm (Dhar and Nandargi, 2004). The state is considered a part of the Eastern Himalayas, a biodiversity hotspot with a geographical area of 82,023 km^2^ and a population density of 17 people per square kilometer (Census, 2011). It is mostly hilly and mountainous, and it is also considered an important eco-region (Olson and Dinerstein, 1998; Bharali and Khan, 2011; Maiti et al., 2017).

Arunachal Pradesh is divided into 25 districts with as many as 26 major tribes and more than 100 sub-tribes in Arunachal Pradesh (Aiyadurai et al., 2009). The art of making Monpa handmade paper originated over 1,000 years ago, and historically, the tradition of making paper is linked to the Buddhist religion and its practices. Gradually, the art became an integral part of local custom and culture in West Kameng and Tawang regions of Arunachal Pradesh.

#### Software and Data Acquisition

ASTER GDEM was downloaded from the Japan Space Systems website^1^ and is utilized to produce the elevation map for the study area. The bioclimatic variables have been taken from the WorldClim database, which is derived from monthly climate data (from 1970 to 2000) for minimum, mean, and maximum temperature, precipitation, solar radiation, wind speed, water vapor pressure, and for total precipitation (Supplementary Table 1). The bioclimatic variables represent annual trends (e.g., mean annual temperature and annual precipitation), seasonality (e.g., annual range in temperature and precipitation), and extreme or limiting environmental factors (e.g., the temperature of the coldest and warmest months and the precipitation of the wet and dry quarters)^2^. To evaluate the potential availability of *D. papyracea*, geographic distribution modeling of the plant species was carried out. The species distribution modeling of *D. papyracea* was performed using the maximum-entropy (maxEnt) technique and the geographical information system (GIS). MaxEnt utilizes a set of known localities, i.e., a set of geographic coordinates where the species has been observed. In addition, data on a number of environmental variables, such as average temperature, average rainfall, elevation, etc., have been measured or estimated across a geographic region of interest.

**FIGURE 2:**
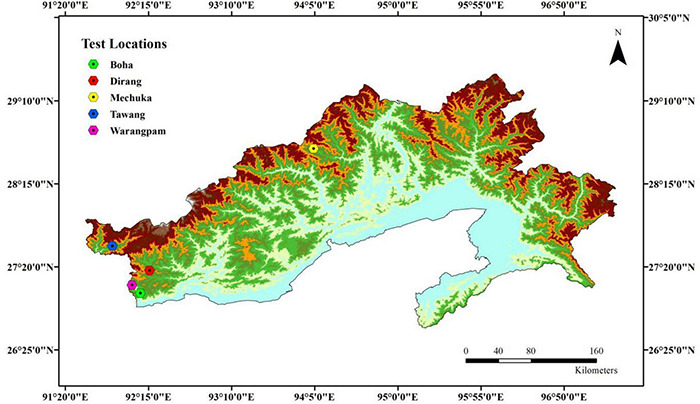
Five known test locations in Arunachal Pradesh where *Daphne papyracea* was seen and used for species distribution modeling.

Preliminary analysis such as masking the layers to the area of interest, modifying the environmental layers to the same extent, etc., was carried out using ArcGIS. The MaxEnt software package 3.4.0 (Phillips et al., 2006, 2020) was used for predicting the species’ occurrence while considering the various environmental variables of known locations. The software generates the probability of species’ occurrence, which ranges from 0 to 1. The 0 and 1 values indicate the lowest and the highest probability of species occurrence, respectively, in a particular geographical area. In this study, five known locations of the species were recorded during a chance encounter while traveling through the state of Arunachal Pradesh (Figure 2). The geographical coordinates, along with the altitudes of the locations, were documented. Nineteen (19) environmental variables (Table 1) with 30-s spatial resolution available in the WorldClim database^3^, along with SRTM elevation, were utilized (Hijmans et al., 2005) to build the Environmental Niche Model and to calibrate the spatial models based on 10-fold cross-validation. It is well established that Maxent performs better at low sample sizes relative to other modeling methods (Bean et al., 2012). We also used the jackknife procedure to generate response curves for each predictor variable and estimate their relative influence. The area under the receiver operating characteristic (ROC) curve, also known as the area under the curve (AUC), was used to evaluate the goodness−of−fit of the model. The model outputs were imported to ArcGIS 10.4 for the final mapping of the suitability distribution of the species. The final output was divided into four suitable habitats that were regrouped with the range of 0–1, *viz*., no suitability (< 0.2); low suitability (0.2–0.4); moderate suitability (0.4–0.6), and high suitability (0.6–1) as per IPCC, 2007.

**TABLE 1:** Environmental variables used in species distribution modeling of *Daphne papyracea* in Arunachal Pradesh.

Environmental variables	Description	Units
Bio1	Annual Mean Temperature	°C
Bio2	Mean Diurnal Range (Mean of monthly maximum and minimum temperature)	°C
Bio3	Isothermality (Bio2/Bio7) (× 100)	–
Bio4	Temperature Seasonality (standard deviation × 100)	C of V
Bio5	Maximum Temperature of Warmest Month	°C
Bio6	Minimum Temperature of Coldest Month	°C
Bio7	Temperature Annual Range (Bio5-Bio6)	°C
Bio8	Mean Temperature of Wettest Quarter	°C
Bio9	Mean Temperature of Driest Quarter	°C
Bio10	Mean Temperature of Warmest Quarter	°C
Bio11	Mean Temperature of Coldest Quarter	°C
Bio12	Annual Precipitation	mm
Bio13	Precipitation of Wettest Month	mm
Bio14	Precipitation of Driest Month	mm
Bio15	Precipitation Seasonality (Coefficient of Variation)	C of V
Bio16	Precipitation of Wettest Quarter	mm
Bio17	Precipitation of Driest Quarter	mm
Bio18	Precipitation of Warmest Quarter	mm
Bio19	Precipitation of Coldest Quarter	mm
Elev	Elevation (SRTM-30s)	m

### Community-Based Production of the Paper From the Bark of *Daphne papyracea*

A community-level traditional handmade paper set up situated in the Tawang region of Arunachal Pradesh was visited for observing the steps and strategies important to producing paper from the bark of the plants. Some important steps involved are discussed below:

**Harvesting or collection:** During this step, mature plants aged 5-6 years are collected and peeled off. The bark is collected, and the wood is used as fuel.

**Scrapping, washing, drying, and soaking:** By using a knife or *dao*, the outer greenish/grayish layers of the bark are scrapped and washed with water to remove the scrapped greenish/grayish layers to obtain the whitish creamy inside part of the bark. The washed bark is then sun-dried for 2 to 3 days and then soaked in water for softening. Scrapping, washing, drying, and soaking are locally called *Khogo*, *Chheyu, Chromo*, and *CheJaso*, respectively.

**Cutting of bark in pieces (*Tapu*)**: In this step, the soaked bark is cut into small pieces, which are further subjected to washing with water to remove dirt and dust if present.

**Boiling:** The cut pieces of bark are boiled in ash water (*BlaPaa*), which is prepared by passing water through ash. About 5 to 6 kilograms of bark are placed in 6 to 8 liters of ash water and boiled for 3-4 h. The boiling is meant to remove any organic substance present. Boiling softens the bark and makes it sticky in texture. To drain the excess water, the boiled bark is placed in a bamboo basket locally called *Sheng Jang*. Boiling of the bark is also termed cooking, and, locally, it is known as *Tsogu*.

**Pulp making or beating:** On a flat stone plate, the boiled fibers are beaten to a pulp with a wooden hammer locally known as *Ruelong.* Then, the uniform paste of fibers is rolled into balls, which allows its easy processing. Beating compresses the pulp, enhances the strength and durability of the paper, and is traditionally referred to as *Thungu*.

**FIGURE 3:**
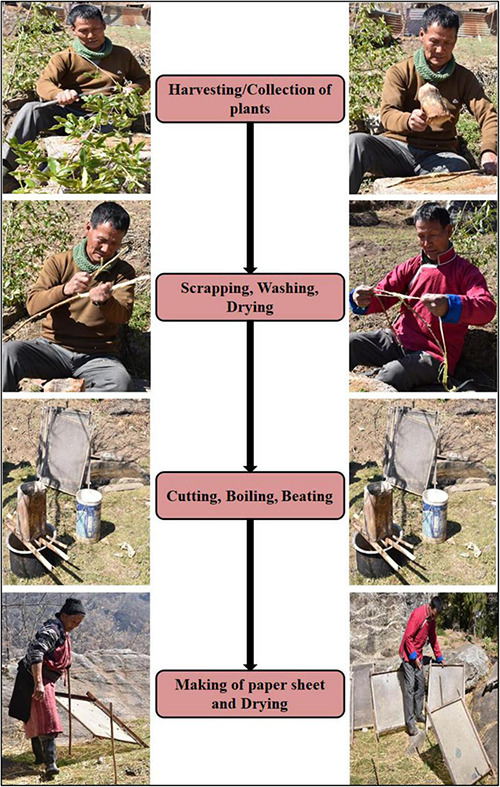
Schematic representation of paper production from the bark of *D. papyracea* in community-based paper making set up at Tawang, Arunachal Pradesh.

**Making of the paper sheet and drying:** The fine pulp is then poured into rectangular frames made up of bamboo wrapped with cloth or galvanized net. The frames are then floated in a water tank and stirred continuously for uniform distribution of the pulp over the net. Making of paper is locally known as *ShukChhusu*, and it requires an aluminum tub (*ShukNema*), a water tank (*Shokang*), a bamboo basket (*Shombu*), and a net (*Soray*). After some time, the frames are carefully taken out of the water tanks and sun-dried for 2 to 3 h. The papers are then peeled off from the frames, collected in bundles, and sold in the local market (Paul et al., 2006). The schematic representation of the steps involved during paper production in a community-based enterprise is shown in Figure 3.

### Glycome Profiling of *Daphne papyracea* Paper

Glycome profiling of *D. papyracea* paper was done in three steps: (a) cell wall preparation, (b) total sugar estimation, and (c) enzyme-linked immunosorbent assay (ELISA) as described earlier (Pattathil et al., 2010, 2012).

**(a) Cell wall preparation:** Alcohol insoluble residue (AIR) was obtained by grinding *D. papyracea* paper and print-copy paper (Office Depot^®^ brand multi-use print and copy paper, United States, item #841195) in liquid nitrogen to fine powder as previously described (Biswal et al., 2015, 2018). Print-copy paper from Office Depot (United States) was used for comparison with *D. papyracea* paper. The powdered biomass was sequentially extracted with 80% (*v*/*v*) ethanol, 100% ethanol, and chloroform/methanol [1:1 (*v*/*v*)]. After centrifugation, the supernatant was discarded, and the resulting cell wall residue (AIR) was air-dried for 72 h at room temperature.

**(b) Total sugar estimation:** A final concentration of 0.2 mg/ml of all the extracts was made by dissolving them in deionized water. The amount of total sugar in all extracts was determined by a phenol-sulfuric acid assay as previously described (Dubois et al., 1951; Masuko et al., 2005; Biswal et al., 2022).

**(c) Enzyme-linked immunosorbent assay:** All wall extract samples were applied (50 μl of 20 μg/ml) to 96-well ELISA plates and dried in an incubator at 37°C for 12 h. The plates were blocked with 200 μl of a blocking buffer to block the non-specific sites in coated ELISA plates and incubated at room temperature for 1 h as described previously (Pattathil et al., 2012). A blocking agent was removed by aspiration, and 50 μl of a primary monoclonal antibody (mAbs) was added to each well and incubated for 1 h at room temperature. Then, each well was washed three times with a 300-μl wash buffer. After washing, 50 μl of a secondary antibody was added to each well and incubated at room temperature for 1 h. The secondary antibodies were washed three times with a 300-μl wash buffer, and 50 μl of TMB (3,3’,5,5’-Tetramethylbenzidine) solution was added to each well. After 20 min, the reaction was stopped by adding 50 μl of 0.5 N sulfuric acid to each well. Finally, the absorbance was measured at 450 nm in an ELISA plate reader (Pattathil et al., 2010, 2012). The results of ELISA were repeated three times with three biological replicates. More detailed descriptions of 155 cell wall glycan-directed monoclonal antibodies are available at the web database, WallMAbDB^4^ and are also provided in the table (Supplementary Table 2).

### Cell Wall Analysis

Prior to the analysis, approximately 40 mg of AIR samples was treated with alpha-amylase (Sigma Cat # A6255) as previously described (Biswal et al., 2017, 2022). Glycosyl residue composition analysis of the AIR (∼ 2 mg) was determined by GC–MS of trimethylsilyl (TMS) derivatization of the monosaccharide methyl glycosides produced from the ground paper by acidic methanolysis as described earlier (Biswal et al., 2015, 2017). All the experiments were repeated three times with three biological replicates.

## Results

### Tradition of Making Handmade Paper Using the Bark of *Daphne papyracea* in Arunachal Pradesh

The art of making Monpa handmade paper has great historic and religious significance as it is the paper used for writing Buddhist scriptures and hymns in monasteries. The practice of writing Buddhist scriptures on handmade paper almost disappeared in the monasteries, and the indigenous handmade paper is taken over by the availability of ready-to-use paper and global modernization. Once produced in every household, this handmade paper was a major source of livelihood for the community, and the monks in monasteries were dependent on handmade paper for writing the sacred texts of teachings of Buddhism, the words of the Buddha, the basis for the teachings of the monks and the birth certificate of a newly born child in a Buddhist family. However, the practice of handmade paper has almost disappeared in the last 100 years, and now, the art of making paper is limited to a few households in the Tawang region of Arunachal Pradesh. It is widely believed that the Buddhist scriptures written on handmade paper are well-preserved without any damage by termites and other insects in the monasteries for many generations. This locally made paper product has great global potential and opens avenues for future research in preservations of age-old scriptures stored in the monasteries.

### Habitat Suitability or Species Distribution Modeling for *Daphne papyracea* in Arunachal Pradesh

**FIGURE 4:**
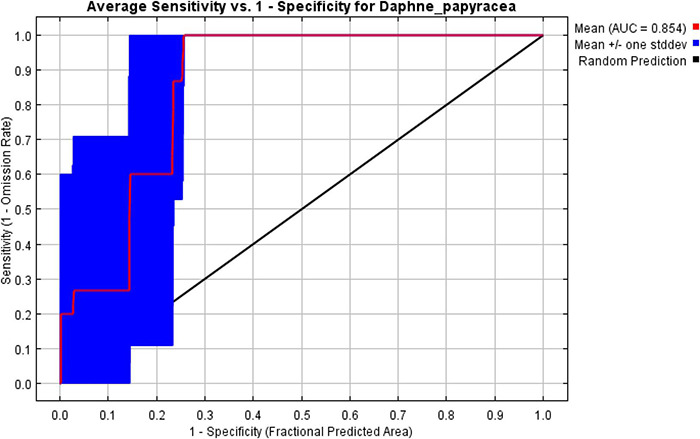
Receiver operating characteristic curve showing sensitivity and specificity of classification.

The goal is to predict which areas within the region satisfy the requirements of the species’ ecological niche, and thus form part of the species’ potential distribution (Anderson and Martinez-Meyer, 2004). The area under the curve (AUC) value of the average model output of the 10-fold cross−validation of the *D. papyracea* was found to be 0.854, combined with a low standard deviation (0.097). The receiver operating characteristic (ROC) is presented in Figure 4. The potential suitable distribution of *D. papyracea* was largely affected by the precipitation variables. Moreover, the precipitation of the driest period (bio14) and isothermality (bio3) were the top two predictors in the Maxent model with contributions of 52.61 and 36.10%, respectively (Table 2). Temperature is another key modulator contributing to the model with varying amounts. For example, the temperature annual range (bio7) and mean temperature of the driest quarter (bio9) have minimally contributed (3.71 and 0.07%, respectively) to determining the distribution of the species. However, temperature seasonality (bio4) has a higher contribution to the model (6.65%).

**TABLE 2:** Variable importance statistics employed in the species distribution modeling of *Daphne papyracea* in Arunachal Pradesh.

Variable	Percent contribution	Importance
bio14	52.61	83.86
bio4	6.65	9.52
bio3	36.10	5.46
bio9	0.07	0.86
bio17	0.83	0.31
bio1	0.00	0.00
bio10	0.00	0.00
bio11	0.00	0.00
bio12	0.00	0.00
bio13	0.00	0.00
bio15	0.00	0.00
bio16	0.00	0.00
bio18	0.00	0.00
bio19	0.00	0.00
bio2	0.00	0.00
bio5	0.05	0.00
bio6	0.00	0.00
bio7	3.71	0.00
bio8	0.00	0.00
Elev	0.00	0.00

**FIGURE 5:**
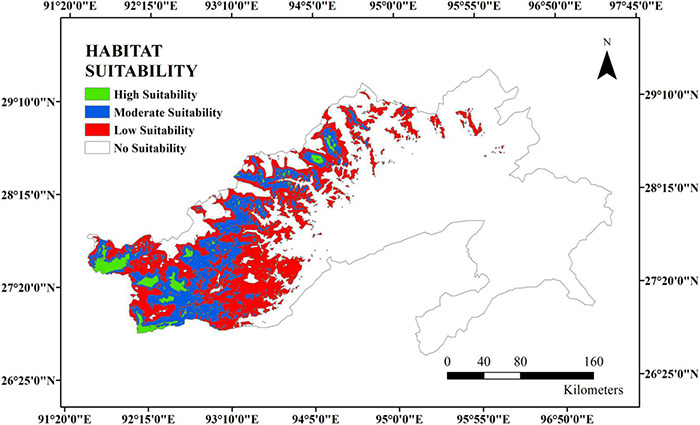
Average final model output of predictive distribution of *D. papyracea* in Arunachal Pradesh.

The predicted suitable habitat of *D. papyracea* shows maximum area distribution toward the western parts of Arunachal Pradesh in general, and the high suitability regions are in the westernmost districts of Arunachal Pradesh, *viz*., West Kameng and Tawang districts (Figure 5). No suitability (68.68%) area was calculated to be the highest in percentage, followed by low suitability (18.87%), moderate suitability (10.41%), and high suitability (2.03%). The higher percentage of the unsuitable area might be a result of a lack of field data such as latitude, longitude, and altitude considered in species distribution modeling. There is a probability that higher sampling and test locations are expected to provide a wide species distribution of *D. papyracea* in different parts of Arunachal Pradesh.

### Production of the Paper From the Bark of the Plant

Figure 3 depicts the process of making handmade paper from the bark of *D. papyracea* using the traditional method. On the basis of information collected and observations from a community-level traditional handmade paper setup, it takes about 5-7 days for paper production, starting from harvesting of the bark up to dispensing and drying of the pulp to produce paper. The handmade production is being carried out at a community level to fulfill the local requirements of making religious prayer flags and Buddhist scripture writings. The processed paper is sold in the market as a pack of 10 pieces at the rate of INR 22 per piece. Although there is no such documentation on the annual production of handmade paper, the *D. papyracea* plant is abundantly found in Arunachal Pradesh to generate raw materials sufficient to meet the increasing demand for handmade paper. *D. papyracea* can regenerate if cut 15 cm above the ground and mature within 4 to 5 years to harvest bark for making paper. *D. papyracea* is a non-grazing, non-timber forest product (NTFP) and not preferred for regular fuel and found growing well under shade and distributed across 1,800-3,000 m altitude.

### Glycome Profiling of Paper Produced From the Bark of the *Daphne papyracea* Plant

**FIGURE 6:**
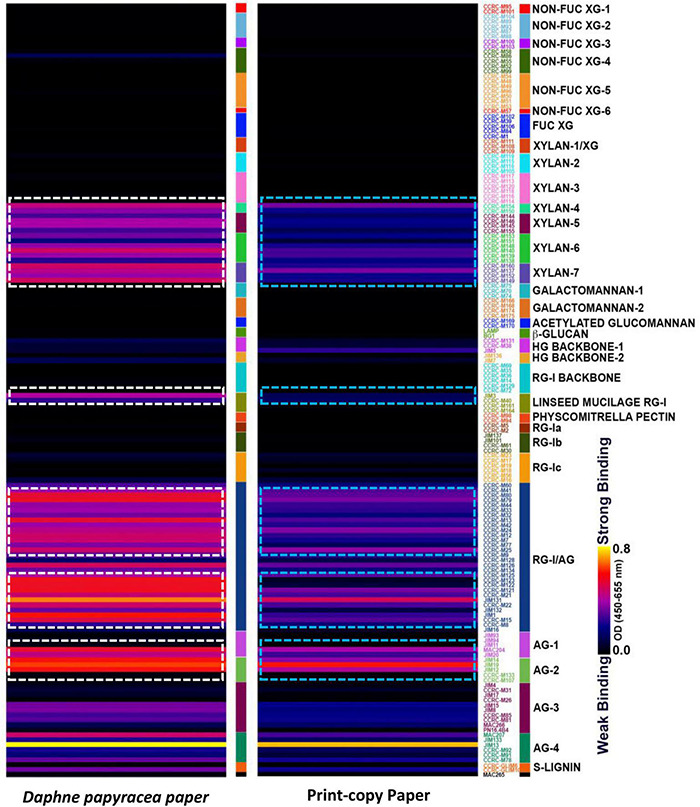
Glycome profile of the handmade paper obtained from the bark of *D. papyracea*. Paper cell walls were prepared from alcohol insoluble residue (AIR). The resulting AIR wall was screened by ELISA using 155 monoclonal antibodies (mAbs) directed against epitopes present on most major non-cellulosic plant cell wall glycans. The mAbs are grouped based on the cell wall glycans they predominantly recognize as depicted in the panel on the right-hand side of the figure. The strength of binding of the mAbs is depicted as a heatmap with bright yellow depicting the strongest binding, dark blue, no binding, and red, intermediate binding. The binding strength of each mAb directly corresponds to the abundance of the specific glycan epitope structure it recognizes, and the differences observed between handmade paper and print copy paper used in this study are shown in the dotted block. Data are the mean of three biological replicates.

To investigate the types of glycans present in the *D. papyracea* paper, we subjected the cell walls (AIR) from *D. papyracea* paper and print-copy paper (Office Depot^®^, United States) to glycome profiling analyses. A set of 155 cell wall glycan-directed monoclonal antibodies (mAbs) was used to screen the wall extracts in this ELISA-based assay (Pattathil et al., 2012). R-Console software was used to present the ELISA responses of these mAbs toward each extract as a heat map. Color gradients are obtained from raw absorbance values with the help of R-Console software. The user chooses the set of color keys for obtaining the color gradients. The binding response data are presented as heatmaps using a dark-blue-red-yellow scale, indicating the strength of the ELISA signal and interpreted as no, medium, and strong bindings, respectively. Glycome profiling data revealed increase in epitope contents in the *D. papyracea* paper compared to print-copy paper. The most consistent changes in *D. papyracea* paper cell wall were observed for xylan backbone epitopes recognized by the Xylan-4, Xylan-5, Xylan-6, and Xylan-7 groups of mAbs. Increased binding of mAbs that specifically bind to linseed mucilage RG-I, RG-I/AG, AG-1, and AG-2 groups was observed in *D. papyracea* paper (white boxes) compared to print-copy paper (green boxes, Figure 6).

### Composition Analyses of Total Cell Walls From *Daphne papyracea* Paper

**FIGURE 7:**
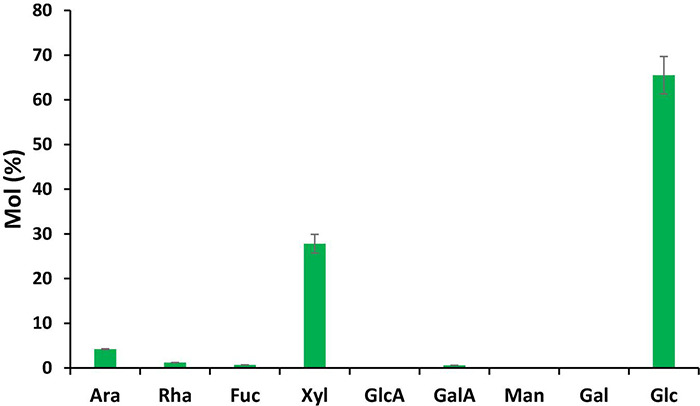
Glycosyl residue composition of alcohol insoluble residue (AIR) from *D. papyracea* paper of tetramethylsilane (TMS) derivatives. The amounts of sugar are represented as average mole%. Data are the mean of three biological replicates.

We investigated the content of cell wall polysaccharides from *D. papyracea* paper. The walls were isolated as alcohol-insoluble residues (AIRs) from handmade paper and analyzed by gas chromatography–mass spectrometry (GC–MS) of trimethylsilyl (TMS) derivatives (Figure 7). A significant amount of 66% of glucose (Glc) and 28% of xylose (Xyl) was observed in the glycosyl residue composition of *D. papyracea* paper. A lower amount of Ara (4%) and Rha (1%) and trace amounts of Fuc and GalA were also observed in the handmade paper.

## Discussion

The history of paper production dates back to the early human civilization and has played a significant role in the economic, social, and environmental development of both developed as well as developing nations. Robust growth in terms of population, literacy, urbanization, and communication has increased the demand for paper production and consumption globally. It has been reported that more than 300 million tons of paper are produced in a single day to meet the high demand of more than 400 million tons throughout the world. Due to the exponential population growth rate, it has been expected that global consumption of paper will reach up to 500 million tons per year by 2025 (Kaur et al., 2017). It has also been documented that, on average, 17 trees are axed down to produce 1 metric ton of paper. Therefore, to meet the growing demand for paper, an excessive number of trees are cut down throughout the world, which results in loss of forest cover, prominent deforestation, and ecosystem degradation. Furthermore, wood-based paper production consumes over 12% of the energy supply in the industry sector, thus qualifying as the third most energy-intensive of all the manufacturing industries. It is reported that 10,000 kWh, 2.57 m^3^, and 25 m^3^ of electricity, oil, and water, respectively, are consumed for the production of 1 metric ton of paper (Alam et al., 2018). Additionally, 220–380 m^3^ of highly colored and potentially toxic wastewater are generated to produce 1 metric ton of wood-based paper, making the paper and pulp industry the fifth largest contributor to water pollution. A total of 500 different chlorinated organics collectively called Adorbable Organic Halides (AOH) are discharged in water bodies, *viz*., chloroform, chlorinated hydrocarbons, syringols, chlorate, resin acids, phenols, furans, catechols, guaiacols, dioxins, vanillin, etc. (Badar and Farooqi, 2012; Haile et al., 2021). Therefore, to meet the increasing global demands for paper, non-timber forest products (NTFPs) or non-wood fibers have turned out to be an important source of fibrous materials for the 21^st^ century (Ashori, 2006). NTFP or non-wood fibrous sources exclusively overcome the resource (wood) shortage and mitigate the environmental issues associated with the paper and pulp industry. The craft of handmade papermaking has been practiced for more than 2,000 years globally. The art flourished and migrated from China to Japan and Korea, and then established in the Islamic World, followed by Europe and America (Hubbe and Bowden, 2009).

Handmade paper production excludes wood as a source of fibrous material, utilizes chemical-free approaches, and focuses on drying up the final products using natural renewable sources (sunlight). Therefore, the handmade practice of papermaking employs non-wood fibrous materials reduces the burden on the decreasing forest cover, stops the release of chemicals to the environment as chemical-free steps are involved during papermaking, and makes the process energy efficient as zero consumption of electricity is encountered during the process. In this regard, the handmade practice of papermaking by the tribes of Arunachal Pradesh from the bark of *D. papyracea* at the community level was studied. The geographic distribution modeling of the plant species was focused to evaluate habitat suitability or species distribution of the plant using the maximum-entropy (maxEnt) technique and GIS (Raina et al., 2014). The potential distribution highlights the regions that have suitable environmental conditions for the species to flourish. This is of great importance as the future viability of the economic exploitation of the species depends on its availability. According to the modeling reports, precipitation variables and temperature are the key modulators of *D. papyracea* distribution. The best-suited habitat was found in the western parts of Arunachal Pradesh, depicting West Kameng and Tawang districts as highly suitable regions. Furthermore, the community-level handmade paper industry in Arunachal Pradesh using the bark of *D. papyracea* was visited, and the whole process of papermaking was observed, starting from the harvesting of plant bark up to the final disposition of beaten soft pulp. Paul et al. (2006) reported that 1-1.5 kg of bark could be obtained from 4-5 plants, and the bark from a single plant could produce two sheets of paper with 62- and 51-cm length and breadth, respectively. The paper is socio-economically important as a large proportion of indigenous people of Arunachal Pradesh are dependent on it for livelihood. Due to its high tensile strength and unique texture, the paper is used for writing Buddhist epics, Mantras, Sutras, Buddhist manuscripts, and temple writings (Paul et al., 2006). The paper is sold in local markets for making gift items and writing purposes. It is pertinent to mention that plant cell walls are composed of a complex polymeric matrix of lignin, cellulose, hemicellulose, pectin, and glycoprotein. The structural integrity of cell walls depends on the covalent and non-covalent linkages between the polymers, structural diversity, and the architectural arrangement between them. The current study revealed that three major classes of cell wall glycans, such as xylan, arabinogalactans, and rhamnogalacturonan-I, are found in the *D. papyracea* paper. Based on this result, we hypothesize that the existence of the age-old Buddhist script on the handmade paper preserved in monasteries is due to the presence of three major classes of glycans (Ryden et al., 2003; Paul et al., 2006). We also performed glycosyl residue composition analysis of the *D. papyracea* paper. Interestingly, a significant amount of glucose and xylose was observed in the *D. papyracea* paper. The saccharide-rich cell walls of plants are reported to perform essential functions, such as maintaining tensile strength and allowing plant growth. Some of the plant-specific monosaccharides are required for the modifications and decoration of several cell wall polysaccharides, including xylan, rhamnogalacturonan I, arabinoxylan, and rhamnogalacturonan II (Perez Garcia et al., 2011). These polysaccharides contribute significantly toward the structural integrity of the cell wall and plant strength. We are hypothesizing that the presence of arabinose, rhamnose, glucose, and xylose maintains structural integrity and enhances the tensile strength of the *D. papyracea* paper. These results suggest that *D. papyracea* raw materials can be used for commercial applications, including paper making, downstream bioproducts, and biomaterial production (Venketachalam et al., 2013).

Owing to its good tensile, double fold, bursting, and tearing strength, the paper could be used for writing, drawing, and printing purposes, for making invitation and greeting cards, paper bags, dairies, paper teacups, biography writings, decorative items, and other fancy products. Due to its multi-utility, vast availability of raw material, environment-friendly nature, biodegradability, etc., it could serve as an alternative to conventional wood-based papers by incorporating innovative ideas to enhance value addition as well as export. Although the handmade paper obtained from the *D. papyracea* bark is eco-friendly, bio-degradable, and recyclable, the handmade papers might not replace the traditional industrial-grade paper due to the absence of modern technical assistance, restricted local use and their production on demand, and a lack of a channel for marketing and export. Khadi and Village Industries Commission (KVIC), Government of Arunachal Pradesh, has commissioned a handmade paper making unit in Tawang to revive the art of making paper, enhancing productivity and empowering the local youth. However, there is an urgent requirement for the incorporation of this indigenous technique of papermaking and the recent innovative approaches to designing different varieties of paper from the bark. A detailed analysis of the paper and its characterization, including whole genome sequencing of plants, may help to enhance the commercial production and value-addition of paper products. Local help groups, NGOs, and government organizations should come forward in creating awareness, educating people about the innovative technique of papermaking, and developing the export market for the substantial socioeconomic development of the people associated with handmade paper making in Arunachal Pradesh.

In conclusion, a significant shift towards non-wood paper production has been reported due to the unavailability of resources (trees) and environmental concerns associated with wood-based pulp and paper industries. Handmade paper enterprises of non-wood resources are regarded as an eco-friendly, cost-effective, energy-efficient, and substantial alternative to the existing wood-based paper industries. In this context, an indigenously prepared paper from the bark of *D. papyracea* was selected for the study. The species distribution modeling of the plant was evaluated for its enhanced accessibility, abundance, and exploration of the scope of future expansion of paper production at a commercial level. For the first time, glycome profiling was done for the plant cell wall characterization and composition of the paper. Finally, ecological integrity, social acceptability, and economic viability are required for uplifting the handmade practice of paper using *D. papyracea* bark.

## Data Availability Statement

The original contributions presented in the study are included in the article/Supplementary Material, further inquiries can be directed to the corresponding author/s.

## Author Contributions

NN, AB, SS, SR, RCD, RD, and MM designed and conceived the experiment. MR wrote the manuscript. NN and AB edited the manuscript. AD helped in data collection and collection of materials. AB performed glycome characterization. NH, VR, KS, and LJ carried out extensive habitat distribution modeling. All authors contributed to the article and approved the submission.

## Conflict of Interest

NN, SS, SR, RCD, and AB have specific interest in detailed documentation, characterization and whole genome sequencing of the paper plant and scientific method of preservation of ancient scriptures of Buddhist script written on hand-made paper in monasteries. The remaining authors declare that the research was conducted in the absence of any commercial or financial relationships that could be construed as a potential conflict of interest.

## Publisher’s Note

All claims expressed in this article are solely those of the authors and do not necessarily represent those of their affiliated organizations, or those of the publisher, the editors and the reviewers. Any product that may be evaluated in this article, or claim that may be made by its manufacturer, is not guaranteed or endorsed by the publisher.

## Footnotes

1
https://ssl.jspacesystems.or.jp/ersdac/GDEM/E/

2
https://www.worldclim.org/data/worldclim21.html

3
www.worldclim.org

4
http://www.wallmabdb.net
