# Supplementary material for: Glycome Profiling and Bioprospecting Potential of the Himalayan Buddhist Handmade Paper of Tawang Region of Arunachal Pradesh
Source: Front Plant Sci. 2022 May 23;13:831589. doi: 10.3389/fpls.2022.831589 (PMC9168884; doi:10.3389/fpls.2022.831589)
Supplement: Supplementary file 1 [file Data_Sheet_1.docx]

Supplementary Material

**Glycome profiling and bioprospecting potential of the Himalayan Buddhist hand-made paper of Tawang region of Arunachal Pradesh**

Muzamil Ahmad Rather^1^, Anutee Dolley^1^, Nabajit Hazarika^2^, Vimha Ritse^3^, Kuladip Sarma^4^, Latonglila Jamir^3^, Siddhartha Shankar Satapathy^5,8^, Suvendra Kumar Ray^1,8^, Ramesh Chandra Deka^6,9^, Ajaya Kumar Biswal^7^, Robin Doley^1^, Manabendra Mandal^1,8^, Nima D. Namsa^1,8,*^

^1^Department of Molecular Biology and Biotechnology, Tezpur University, Napaam 784 028, Assam, India

^2^Department of Environmental Biology and Wildlife Sciences, Cotton University, 781001, Guwahati, Assam, India

^3^Department of Environmental Science, Nagaland University (Central), Lumami-798627, India

^4^Department of Zoology, Cotton University, 781001, Guwahati, Assam, India

^5^Department of Computer Science and Engineering, Tezpur University, Napaam 784 028, Assam, India

^6^Department of Chemical Sciences, Tezpur University, Napaam 784 028, Assam, India

^7^Department of Biochemistry and Molecular Biology and Complex Carbohydrate Research Center, University of Georgia, Athens, GA, United States

^8^Centre for Multi-disciplinary Research, Tezpur University, Napaam 784 028, Assam, India

*****Correspondence: [namsa@tezu.ernet.in (NDN)](mailto:namsa@tezu.ernet.in%20(NDN))

**Supplementary Table 1:** Environmental and its associated variables used in modeling (O Donnell and Ignizio, 2012)

| **Code** | **Description of Environmental variables** | **Unit** |
| --- | --- | --- |
| Bio1 | Annual mean temperature (To determine the annual mean temperature, the climate inputs are averaged over the year.) | ◦C |
| Bio2 | Mean diurnal range (mean of monthly max. and min. temp.) (The mean of the monthly temperature ranges (monthly maximum minus monthly minimum). Since the inputs of climate data are monthly or cumulative months over many years, this calculation uses recorded variations in temperature within a month to capture the diurnal temperature range) | ◦C |
| Bio3 | Isothermality [(Bio2/Bio7) × 100] (It quantifies how large the day-to-night temperatures oscillate compared to the summer-to-winter (annual) oscillations) |  |
| Bio4 | Temperature seasonality (standard deviation ×100)(The rate of temperature variation over a given year (or averaged years) based on the standard deviation (variation) of monthly temperature averages) |  |
| Bio5 | Maximum temperature of warmest month (The maximum monthly temperature occur­rence over a given year (time-series) or averaged year period (normal)) | ◦C |
| Bio6 | Minimum temperature of coldest month (The minimum monthly temperature occur­rence over a given year (time-series) or averaged year period (normal)) | ◦C |
| Bio7 | Temperature annual range (Bio5–Bio6) (A measure of difference in temperatures over a given time) | ◦C |
| Bio8 | Mean temperature of wettest quarter (This quarterly index measures the mean temperatures that persist during the wettest season) | ◦C |
| Bio9 | Mean temperature of driest quarter (This quarterly index measures the mean temperatures that persist during the driest quarter) | ◦C |
| Bio10 | Mean temperature of warmest quarter (This quarterly index measures the mean temperatures that persist during the warmest quarter) | ◦C |
| Bio11 | Mean temperature of coldest quarter (This quarterly index measures the mean temperatures that persist during the coldest quarter) | ◦C |
| Bio12 | Annual precipitation (The total of all total monthly precipitation values) | Mm |
| Bio13 | Precipitation of wettest period (The total precipitation that persist during the wettest month is defined by this index.) | Mm |
| Bio14 | Precipitation of driest period (The total precipitation that persist during the driest month is defined by this index.) | Mm |
| Bio15 | Precipitation seasonality (It measures the variation in monthly precipitation totals over the course of the year. This index is the ratio of the standard deviation of the monthly total precipitation to the mean monthly total precipitation (also known as the coefficient of varia­tion) and is expressed as a percentage) | Mm |
| Bio16 | Precipitation of wettest quarter (This quarterly index calculates the total precipitation that prevails during the wettest quarter) | Mm |
| Bio17 | Precipitation of driest quarter (This quarterly index calculates the total precipitation that prevails during the driest quarter) | Mm |
| Bio18 | Precipitation of warmest quarter (This quarterly index calculates the total precipitation that prevails during the warmest quarter) | Mm |
| Bio19 | Precipitation of coldest quarter (This quarterly index calculates the total precipitation that prevails during the coldest quarter) | Mm |

**Supplementary Table 2: List of cell wall glycan-directed monoclonal antibodies (mAbs) used for glycome profiling analyses.** The groupings of antibodies are based on a hierarchical clustering of ELISA data generated from a screen of all mAbs against a comprehensive panel of plant polysaccharide preparations (Pattathil et al., 2010; Pattathil et al., 2012) that clusters mAbs based on the predominant cell wall glycans that they recognize. The majority of listings link to the Wall*Mab*DB plant cell wall monoclonal antibody database (<http://www.wallmabdb.net>) that provides detailed descriptions of each mAb, including immunogen, antibody isotype, epitope structure (to the extent known), supplier information, and related literature citations.

**Glycan Group Recognized mAb Names**

| Non-Fucosylated Xyloglucan-1 | [CCRC-M95](http://glycomics.ccrc.uga.edu/wall2/jsp/abdetails.jsp?abnumber=162&abname=CCRC-M95) | |
| --- | --- | --- |
|  | [CCRC-M101](http://glycomics.ccrc.uga.edu/wall2/jsp/abdetails.jsp?abnumber=163&abname=CCRC-M101) | |
|  |  | |
|  |  | |
| Non-Fucosylated Xyloglucan-2 | [CCRC-M104](http://glycomics.ccrc.uga.edu/wall2/jsp/abdetails.jsp?abnumber=164&abname=CCRC-M104) | |
|  | [CCRC-M89](http://glycomics.ccrc.uga.edu/wall2/jsp/abdetails.jsp?abnumber=160&abname=CCRC-M89) | |
|  | [CCRC-M93](http://glycomics.ccrc.uga.edu/wall2/jsp/abdetails.jsp?abnumber=161&abname=CCRC-M93) | |
|  | [CCRC-M87](http://glycomics.ccrc.uga.edu/wall2/jsp/abdetails.jsp?abnumber=158&abname=CCRC-M87) | |
|  | [CCRC-M88](http://glycomics.ccrc.uga.edu/wall2/jsp/abdetails.jsp?abnumber=159&abname=CCRC-M88) | |
|  |  | |
|  |  | |
| Non-Fucosylated Xyloglucan-3 | [CCRC-M100](http://glycomics.ccrc.uga.edu/wall2/jsp/abdetails.jsp?abnumber=114&abname=CCRC-M100) | |
|  | [CCRC-M103](http://glycomics.ccrc.uga.edu/wall2/jsp/abdetails.jsp?abnumber=113&abname=CCRC-M103) | |
|  |  | |
|  |  | |
| Non-Fucosylated Xyloglucan-4 | [CCRC-M58](http://glycomics.ccrc.uga.edu/wall2/jsp/abdetails.jsp?abnumber=155&abname=CCRC-M58) | |
|  | [CCRC-M86](http://glycomics.ccrc.uga.edu/wall2/jsp/abdetails.jsp?abnumber=157&abname=CCRC-M86) | |
|  | [CCRC-M55](http://glycomics.ccrc.uga.edu/wall2/jsp/abdetails.jsp?abnumber=148&abname=CCRC-M55) | |
|  | [CCRC-M52](http://glycomics.ccrc.uga.edu/wall2/jsp/abdetails.jsp?abnumber=145&abname=CCRC-M52) | |
|  | [CCRC-M99](http://glycomics.ccrc.uga.edu/wall2/jsp/abdetails.jsp?abnumber=152&abname=CCRC-M99) | |
|  |  | |
|  |  | |
| Non-Fucosylated Xyloglucan-5 | [CCRC-M54](http://glycomics.ccrc.uga.edu/wall2/jsp/abdetails.jsp?abnumber=147&abname=CCRC-M54) | |
|  | [CCRC-M48](http://glycomics.ccrc.uga.edu/wall2/jsp/abdetails.jsp?abnumber=77&abname=CCRC-M48) | |
|  | [CCRC-M49](http://glycomics.ccrc.uga.edu/wall2/jsp/abdetails.jsp?abnumber=76&abname=CCRC-M49) | |
|  | [CCRC-M96](http://glycomics.ccrc.uga.edu/wall2/jsp/abdetails.jsp?abnumber=151&abname=CCRC-M96) | |
|  | [CCRC-M50](http://glycomics.ccrc.uga.edu/wall2/jsp/abdetails.jsp?abnumber=143&abname=CCRC-M50) | |
|  | [CCRC-M51](http://glycomics.ccrc.uga.edu/wall2/jsp/abdetails.jsp?abnumber=144&abname=CCRC-M51) | |
|  | [CCRC-M53](http://glycomics.ccrc.uga.edu/wall2/jsp/abdetails.jsp?abnumber=146&abname=CCRC-M53) | |
|  |  | |
|  |  | |
| Non-Fucosylated Xyloglucan-6 | [CCRC-M57](http://glycomics.ccrc.uga.edu/wall2/jsp/abdetails.jsp?abnumber=154&abname=CCRC-M57) | |
|  |  | |
|  |  | |
| Fucosylated Xyloglucan | [CCRC-M102](http://glycomics.ccrc.uga.edu/wall2/jsp/abdetails.jsp?abnumber=142&abname=CCRC-M102) | |
|  | [CCRC-M39](http://glycomics.ccrc.uga.edu/wall2/jsp/abdetails.jsp?abnumber=78&abname=CCRC-M39) | |
|  | [CCRC-M106](http://glycomics.ccrc.uga.edu/wall2/jsp/abdetails.jsp?abnumber=112&abname=CCRC-M106) | |
|  | [CCRC-M84](http://glycomics.ccrc.uga.edu/wall2/jsp/abdetails.jsp?abnumber=124&abname=CCRC-M84) | |
|  | [CCRC-M1](http://glycomics.ccrc.uga.edu/wall2/jsp/abdetails.jsp?abnumber=1&abname=CCRC-M1) | |
|  |  | |
|  |  | |
| Xylan-1/XG | [CCRC-M111](http://glycomics.ccrc.uga.edu/wall2/jsp/abdetails.jsp?abnumber=168&abname=CCRC-M111) | |
|  | [CCRC-M108](http://glycomics.ccrc.uga.edu/wall2/jsp/abdetails.jsp?abnumber=149&abname=CCRC-M108) | |
|  | [CCRC-M109](http://glycomics.ccrc.uga.edu/wall2/jsp/abdetails.jsp?abnumber=150&abname=CCRC-M109) | |
|  |  | |
|  |  | |
| Xylan-2 | [CCRC-M119](http://glycomics.ccrc.uga.edu/wall2/jsp/abdetails.jsp?abnumber=106&abname=CCRC-M119) | |
|  | [CCRC-M115](http://glycomics.ccrc.uga.edu/wall2/jsp/abdetails.jsp?abnumber=110&abname=CCRC-M115) | |
|  | [CCRC-M110](http://glycomics.ccrc.uga.edu/wall2/jsp/abdetails.jsp?abnumber=167&abname=CCRC-M110) | |
|  | [CCRC-M105](http://glycomics.ccrc.uga.edu/wall2/jsp/abdetails.jsp?abnumber=165&abname=CCRC-M105) | |
|  |  | |
|  |  | |
| Xylan-3 | [CCRC-M117](http://glycomics.ccrc.uga.edu/wall2/jsp/abdetails.jsp?abnumber=108&abname=CCRC-M117) | |
|  | [CCRC-M113](http://glycomics.ccrc.uga.edu/wall2/jsp/abdetails.jsp?abnumber=171&abname=CCRC-M113) | |
|  | [CCRC-M120](http://glycomics.ccrc.uga.edu/wall2/jsp/abdetails.jsp?abnumber=105&abname=CCRC-M120) | |
|  | [CCRC-M118](http://glycomics.ccrc.uga.edu/wall2/jsp/abdetails.jsp?abnumber=107&abname=CCRC-M118) | |
|  | [CCRC-M116](http://glycomics.ccrc.uga.edu/wall2/jsp/abdetails.jsp?abnumber=109&abname=CCRC-M116) | |
|  | [CCRC-M114](http://glycomics.ccrc.uga.edu/wall2/jsp/abdetails.jsp?abnumber=111&abname=CCRC-M114) | |
|  |  | |
| Xylan-4 | CCRC-M154 | |
|  | CCRC-M150 | |
|  |  | |
|  |  | |
| Xylan-5 | CCRC-M144 | |
|  | CCRC-M146 | |
|  | CCRC-M145 | |
|  | CCRC-M155 | |
|  |  | |
|  |  | |
| Xylan-6 | CCRC-M153 | |
|  | CCRC-M151 | |
|  | CCRC-M148 | |
|  | CCRC-M140 | |
|  | CCRC-M139 | |
|  | CCRC-M138 | |
|  |  | |
|  |  | |
| Xylan-7 | CCRC-M160 | |
|  | [CCRC-M137](http://glycomics.ccrc.uga.edu/wall2/jsp/abdetails.jsp?abnumber=173&abname=CCRC-M137) | |
|  | CCRC-M152 | |
|  | CCRC-M149 | |
|  |  | |
|  |  | |
| Galactomannan-1 | [CCRC-M75](http://glycomics.ccrc.uga.edu/wall2/jsp/abdetails.jsp?abnumber=133&abname=CCRC-M75) | |
|  | [CCRC-M70](http://glycomics.ccrc.uga.edu/wall2/jsp/abdetails.jsp?abnumber=61&abname=CCRC-M70) | |
|  | [CCRC-M74](http://glycomics.ccrc.uga.edu/wall2/jsp/abdetails.jsp?abnumber=134&abname=CCRC-M74) | |
|  |  | |
| Galactomannan-2 | CCRC-M166 | |
|  | CCRC-M168 | |
|  | CCRC-M174 | |
|  | CCRC-M175 | |
|  |  | |
|  |  | |
| Glucomannan | CCRC-M169 | |
|  | CCRC-M170 | |
|  |  | |
|  |  | |
| β-Glucan | [LAMP](http://glycomics.ccrc.uga.edu/wall2/jsp/abdetails.jsp?abnumber=47&abname=LAMP2H12H7) | |
|  | [BG1](http://glycomics.ccrc.uga.edu/wall2/jsp/abdetails.jsp?abnumber=48&abname=BG1) | |
|  |  | |
|  |  | |
| HG Backbone-1 | [CCRC-M131](http://glycomics.ccrc.uga.edu/wall2/jsp/abdetails.jsp?abnumber=181&abname=CCRC-M131) | |
|  | [CCRC-M38](http://glycomics.ccrc.uga.edu/wall2/jsp/abdetails.jsp?abnumber=45&abname=CCRC-M38) | |
|  | [JIM5](http://glycomics.ccrc.uga.edu/wall2/jsp/abdetails.jsp?abnumber=14&abname=JIM5) | |
|  |  | |
|  |  | |
| HG Backbone-2 | [JIM136](http://glycomics.ccrc.uga.edu/wall2/jsp/abdetails.jsp?abnumber=57&abname=JIM136) | |
|  | [JIM7](http://glycomics.ccrc.uga.edu/wall2/jsp/abdetails.jsp?abnumber=13&abname=JIM7) | |
|  |  | |
|  |  | |
| RG-I Backbone | [CCRC-M69](http://glycomics.ccrc.uga.edu/wall2/jsp/abdetails.jsp?abnumber=172&abname=CCRC-M69) | |
|  | [CCRC-M35](http://glycomics.ccrc.uga.edu/wall2/jsp/abdetails.jsp?abnumber=66&abname=CCRC-M35) | |
|  | [CCRC-M36](http://glycomics.ccrc.uga.edu/wall2/jsp/abdetails.jsp?abnumber=37&abname=CCRC-M36) | |
|  | [CCRC-M14](http://glycomics.ccrc.uga.edu/wall2/jsp/abdetails.jsp?abnumber=67&abname=CCRC-M14) | |
|  | [CCRC-M129](http://glycomics.ccrc.uga.edu/wall2/jsp/abdetails.jsp?abnumber=104&abname=CCRC-M129) | |
|  | [CCRC-M72](http://glycomics.ccrc.uga.edu/wall2/jsp/abdetails.jsp?abnumber=135&abname=CCRC-M72) | |
|  |  | |
|  |  | |
| Linseed Mucilage RG-I | [JIM3](http://glycomics.ccrc.uga.edu/wall2/jsp/abdetails.jsp?abnumber=79&abname=JIM1) | |
|  | [CCRC-M40](http://glycomics.ccrc.uga.edu/wall2/jsp/abdetails.jsp?abnumber=83&abname=CCRC-M40) | |
|  | CCRC-M161 | |
|  | CCRC-M164 | |
|  |  | |
|  |  | |
| Physcomitrella Pectin | [CCRC-M98](http://glycomics.ccrc.uga.edu/wall2/jsp/abdetails.jsp?abnumber=115&abname=CCRC-M98) | |
|  | [CCRC-M94](http://glycomics.ccrc.uga.edu/wall2/jsp/abdetails.jsp?abnumber=118&abname=CCRC-M94) | |
|  |  | |
|  |  | |
| RG-Ia | [CCRC-M5](http://glycomics.ccrc.uga.edu/wall2/jsp/abdetails.jsp?abnumber=81&abname=CCRC-M5) | |
|  | [CCRC-M2](http://glycomics.ccrc.uga.edu/wall2/jsp/abdetails.jsp?abnumber=8&abname=CCRC-M2) | |
|  |  | |
|  |  | |
| RG-Ib | [JIM137](http://glycomics.ccrc.uga.edu/wall2/jsp/abdetails.jsp?abnumber=58&abname=JIM137) | |
|  | [JIM101](http://glycomics.ccrc.uga.edu/wall2/jsp/abdetails.jsp?abnumber=55&abname=JIM101) | |
|  | [CCRC-M61](http://glycomics.ccrc.uga.edu/wall2/jsp/abdetails.jsp?abnumber=138&abname=CCRC-M61) | |
|  | [CCRC-M30](http://glycomics.ccrc.uga.edu/wall2/jsp/abdetails.jsp?abnumber=33&abname=CCRC-M30) | |
|  |  | |
|  |  | |
| RG-Ic | [CCRC-M23](http://glycomics.ccrc.uga.edu/wall2/jsp/abdetails.jsp?abnumber=92&abname=CCRC-M23) | |
|  | [CCRC-M17](http://glycomics.ccrc.uga.edu/wall2/jsp/abdetails.jsp?abnumber=74&abname=CCRC-M17) | |
|  | [CCRC-M19](http://glycomics.ccrc.uga.edu/wall2/jsp/abdetails.jsp?abnumber=0&abname=CCRC-M19) | |
|  | [CCRC-M18](http://glycomics.ccrc.uga.edu/wall2/jsp/abdetails.jsp?abnumber=0&abname=CCRC-M18) | |
|  | [CCRC-M56](http://glycomics.ccrc.uga.edu/wall2/jsp/abdetails.jsp?abnumber=141&abname=CCRC-M56) | |
|  | [CCRC-M16](http://glycomics.ccrc.uga.edu/wall2/jsp/abdetails.jsp?abnumber=73&abname=CCRC-M16) | |
|  |  | |
|  |  | |
| RG-I/Arabinogalactan | [CCRC-M60](http://glycomics.ccrc.uga.edu/wall2/jsp/abdetails.jsp?abnumber=139&abname=CCRC-M60) | |
|  | [CCRC-M41](http://glycomics.ccrc.uga.edu/wall2/jsp/abdetails.jsp?abnumber=82&abname=CCRC-M41) | |
|  | [CCRC-M80](http://glycomics.ccrc.uga.edu/wall2/jsp/abdetails.jsp?abnumber=128&abname=CCRC-M80) | |
|  | [CCRC-M79](http://glycomics.ccrc.uga.edu/wall2/jsp/abdetails.jsp?abnumber=129&abname=CCRC-M79) | |
|  | [CCRC-M44](http://glycomics.ccrc.uga.edu/wall2/jsp/abdetails.jsp?abnumber=68&abname=CCRC-M44) | |
|  | [CCRC-M33](http://glycomics.ccrc.uga.edu/wall2/jsp/abdetails.jsp?abnumber=75&abname=CCRC-M33) | |
|  | [CCRC-M32](http://glycomics.ccrc.uga.edu/wall2/jsp/abdetails.jsp?abnumber=35&abname=CCRC-M32) | |
|  | [CCRC-M13](http://glycomics.ccrc.uga.edu/wall2/jsp/abdetails.jsp?abnumber=43&abname=CCRC-M13) | |
|  | [CCRC-M42](http://glycomics.ccrc.uga.edu/wall2/jsp/abdetails.jsp?abnumber=86&abname=CCRC-M42) | |
|  | [CCRC-M24](http://glycomics.ccrc.uga.edu/wall2/jsp/abdetails.jsp?abnumber=93&abname=CCRC-M24) | |
|  | [CCRC-M12](http://glycomics.ccrc.uga.edu/wall2/jsp/abdetails.jsp?abnumber=71&abname=CCRC-M12) | |
|  | [CCRC-M7](http://glycomics.ccrc.uga.edu/wall2/jsp/abdetails.jsp?abnumber=3&abname=CCRC-M7) | |
|  | [CCRC-M77](http://glycomics.ccrc.uga.edu/wall2/jsp/abdetails.jsp?abnumber=131&abname=CCRC-M77) | |
|  | [CCRC-M25](http://glycomics.ccrc.uga.edu/wall2/jsp/abdetails.jsp?abnumber=84&abname=CCRC-M25) | |
|  | [CCRC-M9](http://glycomics.ccrc.uga.edu/wall2/jsp/abdetails.jsp?abnumber=69&abname=CCRC-M9) | |
|  | [CCRC-M128](http://glycomics.ccrc.uga.edu/wall2/jsp/abdetails.jsp?abnumber=183&abname=CCRC-M128) | |
|  | [CCRC-M126](http://glycomics.ccrc.uga.edu/wall2/jsp/abdetails.jsp?abnumber=184&abname=CCRC-M126) | |
|  | [CCRC-M134](http://glycomics.ccrc.uga.edu/wall2/jsp/abdetails.jsp?abnumber=102&abname=CCRC-M134) | |
|  | [CCRC-M125](http://glycomics.ccrc.uga.edu/wall2/jsp/abdetails.jsp?abnumber=185&abname=CCRC-M125) | |
|  | [CCRC-M123](http://glycomics.ccrc.uga.edu/wall2/jsp/abdetails.jsp?abnumber=187&abname=CCRC-M123) | |
|  | [CCRC-M122](http://glycomics.ccrc.uga.edu/wall2/jsp/abdetails.jsp?abnumber=188&abname=CCRC-M122) | |
|  | [CCRC-M121](http://glycomics.ccrc.uga.edu/wall2/jsp/abdetails.jsp?abnumber=189&abname=CCRC-M121) | |
|  | [CCRC-M112](http://glycomics.ccrc.uga.edu/wall2/jsp/abdetails.jsp?abnumber=169&abname=CCRC-M112) | |
|  | [CCRC-M21](http://glycomics.ccrc.uga.edu/wall2/jsp/abdetails.jsp?abnumber=88&abname=CCRC-M21) | |
|  | [JIM131](http://glycomics.ccrc.uga.edu/wall2/jsp/abdetails.jsp?abnumber=94&abname=JIM131) | |
|  | [CCRC-M22](http://glycomics.ccrc.uga.edu/wall2/jsp/abdetails.jsp?abnumber=46&abname=CCRC-M22) | |
|  | [JIM132](http://glycomics.ccrc.uga.edu/wall2/jsp/abdetails.jsp?abnumber=56&abname=JIM132) | |
|  | [JIM1](http://glycomics.ccrc.uga.edu/wall2/jsp/abdetails.jsp?abnumber=79&abname=JIM1) | |
|  | [CCRC-M15](http://glycomics.ccrc.uga.edu/wall2/jsp/abdetails.jsp?abnumber=72&abname=CCRC-M15) | |
|  | [CCRC-M8](http://glycomics.ccrc.uga.edu/wall2/jsp/abdetails.jsp?abnumber=29&abname=CCRC-M8) | |
|  | [JIM16](http://glycomics.ccrc.uga.edu/wall2/jsp/abdetails.jsp?abnumber=62&abname=JIM16) | |
|  |  | |
|  |  | |
| Arabinogalactan-1 | [JIM93](http://glycomics.ccrc.uga.edu/wall2/jsp/abdetails.jsp?abnumber=117&abname=JIM93) | |
|  | [JIM94](http://glycomics.ccrc.uga.edu/wall2/jsp/abdetails.jsp?abnumber=95&abname=JIM94) | |
|  | [JIM11](http://glycomics.ccrc.uga.edu/wall2/jsp/abdetails.jsp?abnumber=41&abname=JIM11) | |
|  | [MAC204](http://glycomics.ccrc.uga.edu/wall2/jsp/abdetails.jsp?abnumber=23&abname=MAC204) | |
|  | [JIM20](http://glycomics.ccrc.uga.edu/wall2/jsp/abdetails.jsp?abnumber=91&abname=JIM20) | |
|  |  | |
|  |  | |
| Arabinogalactan-2 | [JIM14](http://glycomics.ccrc.uga.edu/wall2/jsp/abdetails.jsp?abnumber=31&abname=JIM14) | |
|  | [JIM19](http://glycomics.ccrc.uga.edu/wall2/jsp/abdetails.jsp?abnumber=44&abname=JIM19) | |
|  | [JIM12](http://glycomics.ccrc.uga.edu/wall2/jsp/abdetails.jsp?abnumber=191&abname=JIM12) | |
|  | [CCRC-M133](http://glycomics.ccrc.uga.edu/wall2/jsp/abdetails.jsp?abname=CCRC-M133) | |
|  | [CCRC-M107](http://glycomics.ccrc.uga.edu/wall2/jsp/abdetails.jsp?abnumber=166&abname=CCRC-M107) | |
|  |  | |
|  |  | |
| Arabinogalactan-3 | [JIM4](http://glycomics.ccrc.uga.edu/wall2/jsp/abdetails.jsp?abnumber=40&abname=JIM4) | |
|  | [CCRC-M31](http://glycomics.ccrc.uga.edu/wall2/jsp/abdetails.jsp?abnumber=34&abname=CCRC-M31) | |
|  | [JIM17](http://glycomics.ccrc.uga.edu/wall2/jsp/abdetails.jsp?abnumber=39&abname=JIM17) | |
|  | [CCRC-M26](http://glycomics.ccrc.uga.edu/wall2/jsp/abdetails.jsp?abnumber=85&abname=CCRC-M26) | |
|  | [JIM15](http://glycomics.ccrc.uga.edu/wall2/jsp/abdetails.jsp?abnumber=32&abname=JIM15) | |
|  | [JIM8](http://glycomics.ccrc.uga.edu/wall2/jsp/abdetails.jsp?abnumber=80&abname=JIM8) | |
|  | [CCRC-M85](http://glycomics.ccrc.uga.edu/wall2/jsp/abdetails.jsp?abnumber=121&abname=CCRC-M85) | |
|  | [CCRC-M81](http://glycomics.ccrc.uga.edu/wall2/jsp/abdetails.jsp?abnumber=127&abname=CCRC-M81) | |
|  | [MAC266](http://glycomics.ccrc.uga.edu/wall2/jsp/abdetails.jsp?abnumber=98&abname=MAC266) | |
|  | [PN 16.4B4](http://glycomics.ccrc.uga.edu/wall2/jsp/abdetails.jsp?abnumber=11&abname=PN%2016.4B4) | |
|  |  | |
|  |  | |
| Arabinogalactan-4 | [MAC207](http://glycomics.ccrc.uga.edu/wall2/jsp/abdetails.jsp?abnumber=22&abname=MAC207) | |
|  | [JIM133](http://glycomics.ccrc.uga.edu/wall2/jsp/abdetails.jsp?abnumber=96&abname=JIM133) | |
|  | [JIM13](http://glycomics.ccrc.uga.edu/wall2/jsp/abdetails.jsp?abnumber=30&abname=JIM13) | |
|  | [CCRC-M92](http://glycomics.ccrc.uga.edu/wall2/jsp/abdetails.jsp?abnumber=119&abname=CCRC-M92) | |
|  | [CCRC-M91](http://glycomics.ccrc.uga.edu/wall2/jsp/abdetails.jsp?abnumber=120&abname=CCRC-M91) | |
|  | [CCRC-M78](http://glycomics.ccrc.uga.edu/wall2/jsp/abdetails.jsp?abnumber=130&abname=CCRC-M78) | |
|  |  | |
| S-LIGNIN | [CCRC-GLIM6](http://glycomics.ccrc.uga.edu/wall2/jsp/abdetails.jsp?abnumber=81&abname=CCRC-M5) | |
|  | [CCRC-GLIM10](http://glycomics.ccrc.uga.edu/wall2/jsp/abdetails.jsp?abnumber=8&abname=CCRC-M2) | |
|  |  | |
|  |  | |
| Unidentified | [MAC265](http://glycomics.ccrc.uga.edu/wall2/jsp/abdetails.jsp?abnumber=97&abname=MAC265) |  |
|  |  |  |
|  |  |  |
|  |  | |
|  |  | |

**References**

1. O Donnell, M.S., Ignizio, D.A. 2012. Bioclimatic predictors for supporting ecological applications in the conterminous United States: U.S. Geological Survey Data Series 691, 10 p.

2. Pattathil S, Avci U, Baldwin D et al. A comprehensive toolkit of plant cell wall glycan-directed monoclonal antibodies. Plant Physiol 2010; 153:514-525.

3. Pattathil S, Avci U, Hahn MG Immunological approaches to plant cell wall and biomass characterization: glycome profiling. Methods Mol Biol 2012, 908: 61-72.
